# Sporadic DUX4 expression in FSHD myocytes is associated with incomplete repression by the PRC2 complex and gain of H3K9 acetylation on the contracted D4Z4 allele

**DOI:** 10.1186/s13072-018-0215-z

**Published:** 2018-08-20

**Authors:** Premi Haynes, Karol Bomsztyk, Daniel G. Miller

**Affiliations:** 1 Departments of Pediatrics and Genome Sciences, University of Washington, Seattle, WA USA; 2 Department of Medicine, University of Washington, Seattle, WA USA; 3 University of Washington, Campus Box 358056, 850 Republican Street, Room N416, Seattle, WA 98109 USA

**Keywords:** FSHD, Facioscapulohumeral, Muscular, Dystrophy, DUX4, Epigenetics, Chromatin, Histone, D4Z4, Neuromuscular, SMCHD1, H3K9Ac, PRC2, Polycomb repressive complex, Bivalent

## Abstract

**Background:**

Facioscapulohumeral muscular dystrophy 1 (FSHD1) has an autosomal dominant pattern of inheritance and primarily affects skeletal muscle. The genetic cause of FSHD1 is contraction of the D4Z4 macrosatellite array on chromosome 4 alleles associated with a permissive haplotype causing infrequent sporadic expression of the DUX4 gene. Epigenetically, the contracted D4Z4 array has decreased cytosine methylation and an open chromatin structure. Despite these genetic and epigenetic changes, the majority of FSHD myoblasts are able to repress DUX4 transcription. In this study we hypothesized that histone modifications distinguish DUX4 expressing and non-expressing cells from the same individuals.

**Results:**

FSHD myocytes containing the permissive 4qA haplotype with a long terminal D4Z4 unit were sorted into DUX4 expressing and non-expressing groups. We found similar CpG hypomethylation between the groups of FSHD-affected cells suggesting that CpG hypomethylation is not sufficient to trigger DUX4 expression. A survey of histone modifications present at the D4Z4 region during cell lineage commitment revealed that this region is bivalent in FSHD iPS cells with both H3K4me3 activating and H3K27me3 repressive marks present, making D4Z4 poised for DUX4 activation in pluripotent cells. After lineage commitment, the D4Z4 region becomes univalent with H3K27me3 in FSHD and non-FSHD control myoblasts and a concomitant increase in H3K4me3 in a small fraction of cells. Chromatin immunoprecipitation (ChIP) for histone modifications, chromatin modifier proteins and chromatin structural proteins on sorted FSHD myocytes revealed that activating H3K9Ac modifications were ~ fourfold higher in DUX4 expressing FSHD myocytes, while the repressive H3K27me3 modification was ~ fourfold higher at the permissive allele in DUX4 non-expressing FSHD myocytes from the same cultures. Similarly, we identified EZH2, a member of the polycomb repressive complex involved in H3K27 methylation, to be present more frequently on the permissive allele in DUX4 non-expressing FSHD myocytes.

**Conclusions:**

These results implicate PRC2 as the complex primarily responsible for DUX4 repression in the setting of FSHD and H3K9 acetylation along with reciprocal loss of H3K27me3 as key epigenetic events that result in DUX4 expression. Future studies focused on events that trigger H3K9Ac or augment PRC2 complex activity in a small fraction of nuclei may expose additional drug targets worthy of study.

**Electronic supplementary material:**

The online version of this article (10.1186/s13072-018-0215-z) contains supplementary material, which is available to authorized users.

## Background

Facioscapulohumeral muscular dystrophy 1 (FSHD1) has an autosomal dominant pattern of inheritance [1] and manifests as a consequence of both genetic [2–4] and epigenetic disease mechanisms [5]. FSHD is most commonly present in the second decade of life as asymmetric weakness of specific skeletal or facial muscle groups [6]. Regardless of the genetic mechanism, FSHD results from abnormal expression of double homeobox protein 4 (DUX4) in skeletal muscle [7]. The DUX4 gene is encoded in each of the 3.3 kb D4Z4 repeat units arrayed at chromosome 4q35.

DUX4 expression is important early in development when it activates ZSCAN4 as part of a chromatin remodeling phase that occurs in 4 cell embryos [8] and in most adult tissues DUX4 transcription is strongly repressed. A recent proteomics-based study identified proteins that specifically bind to the D4Z4 array and showed that nucleosome remodeling deacetylase (NuRD) and CAF-1 complexes repress DUX4 transcription in control myoblasts and induced pluripotent stem cells [9]. The D4Z4 array length appears to be critical for DUX4 repression because FSHD-causing array contractions result in toxic DUX4 expression when they occur on a common 4q haplotype that includes a polyadenylation signal at the end of the last D4Z4 unit [7, 10, 11]. Array length is not the only mediator of transcriptional repression because even in the context of an array contraction, DUX4 remains under significant although incomplete repression with cultured myoblasts showing stochastic bursts of transcription in a small fraction of myonuclei [12–14].

Differences in histone modifications associated with FSHD-causing contracted and normal length D4Z4 arrays have been difficult to identify because of the presence of multiple D4Z4 units near the telomere of chromosome 10, the normal D4Z4 array on the other allele of chromosome 4 and D4Z4-like sequences at multiple other genomic locations [15]. As minor sequence differences of these arrays have been catalogued, primers with some specificity for D4Z4 units originating from chromosomes 4 and 10 have been developed [16] and despite averaging PCR signals originating from each repeat on chromosomes 4 and 10 and from D4Z4 units within both pathogenic and non-pathogenic arrays, a decrease in the levels of H3K9me3 and 5-meC D4Z4 modifications [16] and a decrease in cohesion and HP1γ association with D4Z4 have been observed in cells derived from FSHD-affected individuals [17]. More recently, a bisulfite DNA sequencing approach has allowed methylation levels to be attributed to specific alleles identified by single nucleotide polymorphisms present in the sequenced regions and has convincingly demonstrated that CpG hypomethylation is localized to contracted arrays in cells from FSHD1-affected individuals [18]. The ability to specifically characterize differences in histone modifications present on DUX4-expressing and non-expressing contracted D4Z4 arrays on chromosome 4 should reveal new and important epigenetic differences associated with FSHD.

To determine the epigenetic changes that result in DUX4 transcription, we took advantage of the observation that FSHD-permissive 4qA alleles are polymorphic with two common haplotypes that differ in the length of the terminal D4Z4 unit [3]. We also constructed and validated a DUX4 reporter that allows the detection and collection of DUX4 expressing cells using GFP fluorescence [13]. This approach allows pathogenic (contracted) arrays to be specifically analyzed in the presence of other arrays in the same cells and allows comparisons of epigenetic differences present at D4Z4 in DUX4 expressing and non-expressing cells from the same myoblast population.

By measuring the frequency of association of chromatin remodeling proteins and the abundance of epigenetic marks present on DNA and histones at D4Z4 arrays, we were able to follow the epigenetic state of normal and pathogenic D4Z4 arrays in stem cells and terminally differentiated human myocytes. Importantly, we were able to distinguish epigenetic differences present at DUX4 expressing and DUX4 non-expressing pathogenic arrays within the same myocyte population. We show that all D4Z4 arrays are bivalent with respect to repressive and activating histone modifications in stem cells and thus poised for DUX4 expression, consistent with the observation of DUX4 expression at the 4 cell embryonic stage where DUX4 appears to be instrumental in establishing the epigenetic profile of the early embryo [8]. As stem cells commit to terminally differentiated states, long arrays adopt a repressive chromatin confirmation while histones on short arrays are left vulnerable to further modifications including the removal of H3K27me3 and acetylation of H3K9 leading to the activation of DUX4 transcription.

## Results

### Exclusive detection of pathogenic alleles produces an enhanced signal for analysis of FSHD-associated D4Z4 chromatin structure

**Fig. 1:**
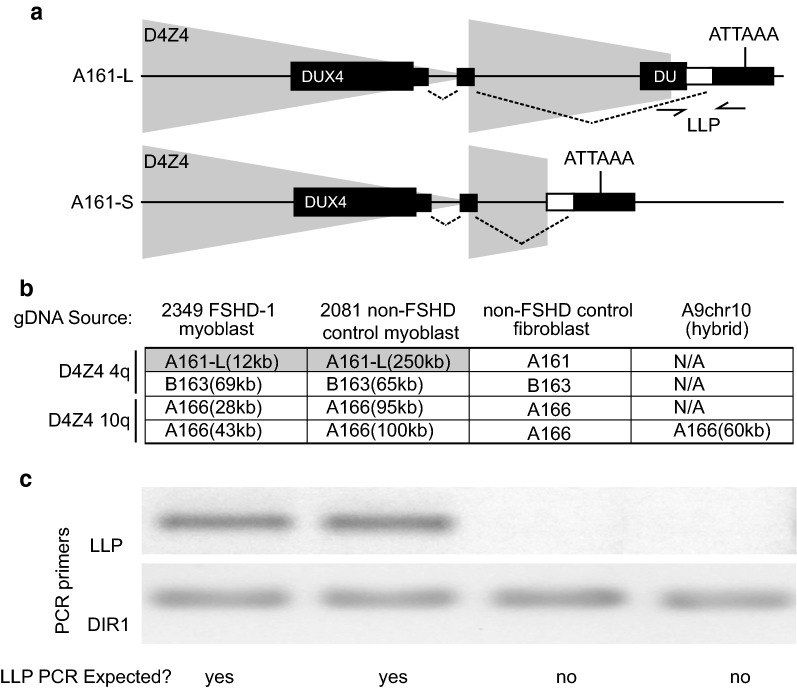
PCR amplification of permissive alleles in FSHD cells. **a** Diagram of two unique terminal D4Z4 junctions of permissive alleles in FSHD. Both haplotypes contain a permissive SSLP of 161 bp length and a polyadenylation signal (ATTAAA). The junction of the distal end of the D4Z4 array is different resulting in a slightly longer 3′ untranslated region in the A161-L version [also called long last partial (LLP)]. Introns are noted by dashed lines between splice sites and exons are noted as black boxes with the thicker portion corresponding to the ORF of the DUX4 protein. PCR primers that uniquely identify the A161-L form are shown as arrows in the distal region of the array (LLP). **b** Table showing genotypes of the cell lines used to characterize the LLP primers. DIR1 primers are homologous to a common region present in all arrays just centromeric to the start. Note that FSHD1 and non-FSHD control myoblast lines both have permissive A161-L arrays with disease causing length of 12 kb in the FSHD line (2 repeats) and disease protective length in the control line (74 repeats). **c** DNA fragments amplified from genomic DNA purified from the cell lines shown in (**b**) and the expected result shown below. The locations of the LLP primers are shown in (**a**)

The contraction of the D4Z4 array associated with the allele variant 4qA is required to cause FSHD1 [7, 19]. There are two sub-haplotypes of 4qA that contain a permissive SSLP of 161 length associated with a poly A signal sequence at the distal end of the array [7]. The sub-haplotypes differ at the distal most D4Z4 unit where the haplotype A161-L (Fig. 1a) contains an additional ~2 kb of non-translated D4Z4 sequence. Since this represents a different breakpoint in the most telomeric D4Z4 unit which is always partially present, we call this variant the long last partial (LLP) to distinguish it from the more common last partial D4Z4 unit that is shorter due to a breakpoint earlier in the last D4Z4 unit (SLP). Individuals with FSHD1 can have either a LLP or SLP sub-haplotype [3] as both are permissive and associated with the poly A signal sequence. We utilized custom PCR primers that specifically amplify the A161-L but not the A161-S on 4qA alleles (Fig. 1b). The control and FSHD1 myoblasts utilized in this study were PCR amplified with these LLP primers and identified to have the 4q A161-L haplotype (Fig. 1c). The DUX4-interacting region 1 (DIR1) a region proximal to the LLP and common to all the genotypes studied [20] was amplified with a separate set of primers as a control. We screened several myoblast cell lines to identify those that have a single permissive A-type array with a LLP sub-haplotype allowing us to specifically probe the chromatin structure of the DUX4 expressing array in cells from FSHD-affected individuals.

### DUX4 expressing and non-expressing FSHD myocytes are similarly hypomethylated on the pathogenic D4Z4 array

**Fig. 2:**
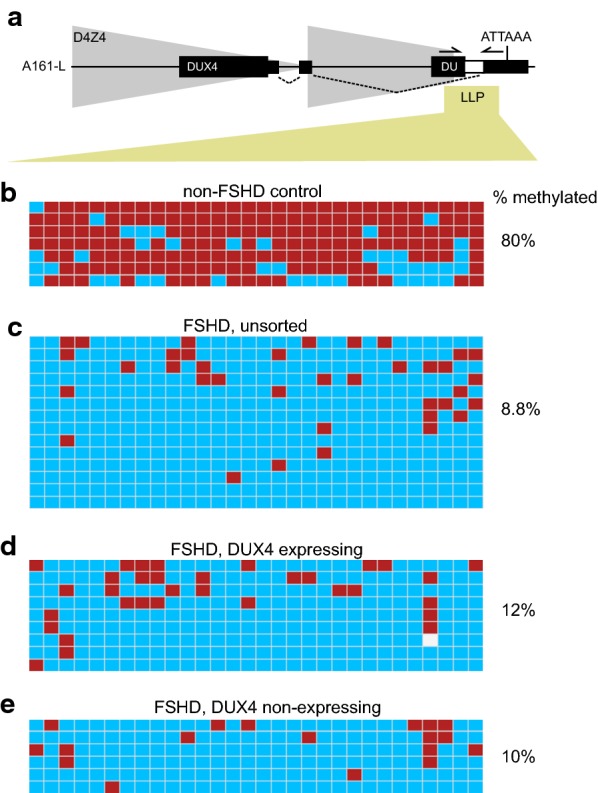
CpG methylation density from DUX4 expressing and DUX4 non-expressing arrays in FSHD and non-FSHD control myocytes. CpG methylation events are shown from sorted populations of bisulfite-treated non-FSHD control or FSHD-affected differentiated myocytes. Primers that uniquely amplify the 4qA-161-L haplotype were used so only methylation events from the 4q A161-L haplotype are shown. **a** Diagram of a full D4Z4 unit and terminal D4Z4 partial unit (LLP) with portions of full length (DUX4) or partial (DU) DUX4 genes shown as black rectangles. The position of LLP PCR primers are shown as converging arrows. **b** CpG methylation pattern in non-FSHD control myocytes containing a single LLP D4Z4 array (Fig. 1, 2081). **c** CpG methylation pattern of the LLP region in unsorted FSHD-affected differentiated myocytes (Fig. 1, 2349). **d** CpG methylation pattern of the LLP region in the DUX4 expressing population of cells from FSHD-affected differentiated myocytes (Fig. 1, 2349). **e** CpG methylation pattern of the LLP region in the DUX4 non-expressing population of cells from FSHD-affected differentiated myocytes (Fig. 1, 2349). The percentage of CpG methylation are indicated to the right-hand side for each group. The location of methylated cytosines is shown as red squares, and the location of unmethylated cytosines are shown as blue squares. DNA variants which result in a sequence but are no longer a CpG are colored white

To determine whether DUX4 transcription is repressed by DNA methylation in FSHD myoblasts that don’t express DUX4, we investigated basal CpG methylation levels at the permissive, contracted array using bisulfite conversion and PCR primers that uniquely amplify the LLP allele [21]. D4Z4 in normal myocytes containing a LLP allele was 80% methylated, while the same region was only 8.8% methylated in myocytes from FSHD-affected individuals supporting previously published data (Fig. 2b, c) [5, 22]. Utilizing a fluorescing DUX4 reporter, we purified myocytes that expressed DUX4 from those that efficiently silenced DUX4 and show that myoblasts express 0.26–1.09% DUX4 and when differentiated the myocytes express 1.23–3.75% DUX4 (Additional file 1: Figure S1). These results suggest that even under optimal culture conditions, only a fraction of cells are expressing DUX4, suggesting that DUX4 repression is robust even in the disease state [13]. We quantified LLP methylation levels by bisulfite conversion of genomic DNA from DUX4 expressing and DUX4 non-expressing cell populations. CpG methylation levels were similar whether the cells contained arrays that expressed DUX4 (12% methylation) or contained transcriptionally silent arrays (10% methylation) (Fig. 2d, e). Thus, even though the pathogenic array is hypomethylated in all FSHD myocytes, CpG methylation levels are not significantly different in DUX4-expressing and DUX4 non-expressing myocyte populations suggesting that additional epigenetic signals are responsible for the sporadic DUX4 expression from pathogenic arrays in FSHD.

### The D4Z4 array has a bivalent chromatin signature in stem cells that becomes univalent and associated with H3K27me3 labeled histones in FSHD myoblasts

**Fig. 3:**
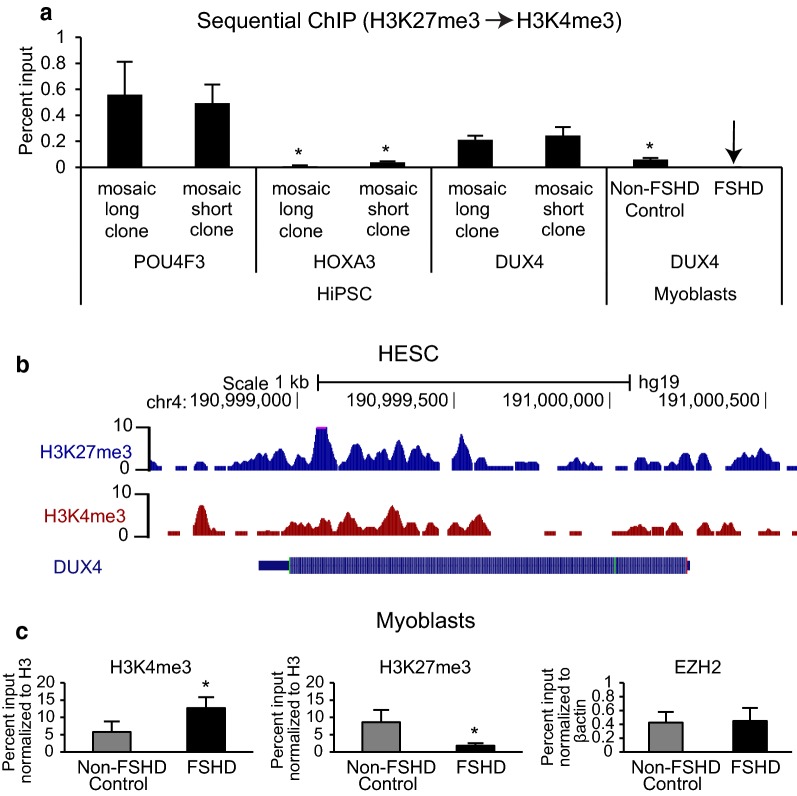
The D4Z4 locus is bivalent in human stem cells and turns univalent in myoblast. **a** Sequential chromatin immunoprecipitation beginning with antibodies to H3K27me3 and followed by antibodies to H3K4me3 was utilized to investigate bivalency at the DUX4 locus. The reverse sequential ChIP with antibodies recognizing H3K4me3 first and antibodies recognizing H3K27me3s was also performed with similar results (data not shown). Isogenic human iPS cell clones isolated from individuals with mosaic distributions of D4Z4 lengths were used for this study. Chromatin purified from iPS clones with non-contracted (mosaic long clone) and contracted (mosaic short clone) iPS cell clones was utilized in ChIP and quantitative PCR amplification. The stem cell bivalent locus (POU4F3) [25] and the stem cell univalent locus (HOXA3) [24, 25] were utilized as controls for comparison to the LLP region of D4Z4 in the iPS cell clones. Similarly, sequential ChIP was performed on myoblasts from non-FSHD control and FSHD myoblasts again uniquely amplifying the 4qA161-L allele in these cells. The arrow indicates signal was not detected. The pull down for the POU4F3 locus in the mosaic long clone was statistically significant using the Student’s *t* test when compared to the HOXA3 locus in the mosaic long clone and the DUX4 locus in the normal primary myoblast (*p value ≤ 0.05). Similarly, the pull down for POU4F3 locus in mosaic short clone was significantly different when compared to the HOXA3 locus in the mosaic short clone (*p value ≤ 0.05). **b** ChIP-seq data [25] were reprocessed and aligned to the human genome for DUX4 analysis. Similar levels of H3K27me3 and H3K4me3 at the DUX4 locus supports a bivalent chromatin structure and is consistent with our ChIP results in (**a**). **c** Chromatin immunoprecipitation of D4Z4 DNA with antibodies recognizing H3K4me3, H3K27me3, an EZH2 and selective amplification of the permissive allele in non-FSHD control and FSHD1 myoblasts. Comparison by *t* test of the mean percent input normalized to H3 between non-FSHD control to FSHD for H3K4me3 and H3K27me3 revealed statistical significance (*p value ≤ 0.05). The error bars show standard deviations

DUX4 has sequence homology to several homeotic transcription factors that determine cell fate early in development and most closely resembles homeotic transcription factors of the Paired class [23]. A recurring regulatory theme among these developmentally important genes is the observation that early in development the chromatin structure is composed of a mixture of histones containing repressive modifications (H3K27me3) and activating modifications (H3K4me3) [24]. These bivalent loci are thought to be poised to commit to silencing or expression depending on the cell fate choice. We used antibodies specific for H3K4me3 or H3K27me3 to immunoprecipitate chromatin from isogenic human iPS cells cloned from the normal and contracted D4Z4 populations present in an individual with FSHD who was mosaic for D4Z4 array size and contained a 4qA-L sub-haplotype. Single ChIP pull down with either H3K4me3 or H3K27me3 revealed that the isogenic human iPS mosaic clones had both marks present individually (Additional file 1: Figure S2). Further analysis showed that like the known bivalent locus POU4F3, the D4Z4 contracted and non-contracted stem cell clones contained both H3K4me3 and H3K27me3 labeled histones at D4Z4 and sequential ChIP experiments [24] showed the marks were present at the same locus in the same cells confirming that D4Z4 is bivalent in stem cell populations (Fig. 3a). Re-examination of previously published ChIP-seq data [25] revealed bivalent histone modifications in several human ES cell lines as well (Fig. 3b). However, fully committed primary myoblasts from FSHD muscle biopsies contained univalent D4Z4 loci yielding almost undetectable signal after sequential immunoprecipitation similar to the known univalent locus HOXA3 (Fig. 3a). We also confirmed previous observations [17] that the D4Z4 array in myoblasts is labeled with H3K27me3 in affected and unaffected individuals using conventional immunoprecipitation (Fig. 3c). Myoblasts from FSHD-affected individuals did have higher H3K4me3 modification levels when compared to non-FSHD primary myoblasts from controls consistent with the presence of transcriptionally active arrays in some cells of the FSHD-affected population (Fig. 3c).

### The pathogenic D4Z4 array contains H3K9 acetylated histones in DUX4 expressing cells

**Fig. 4:**
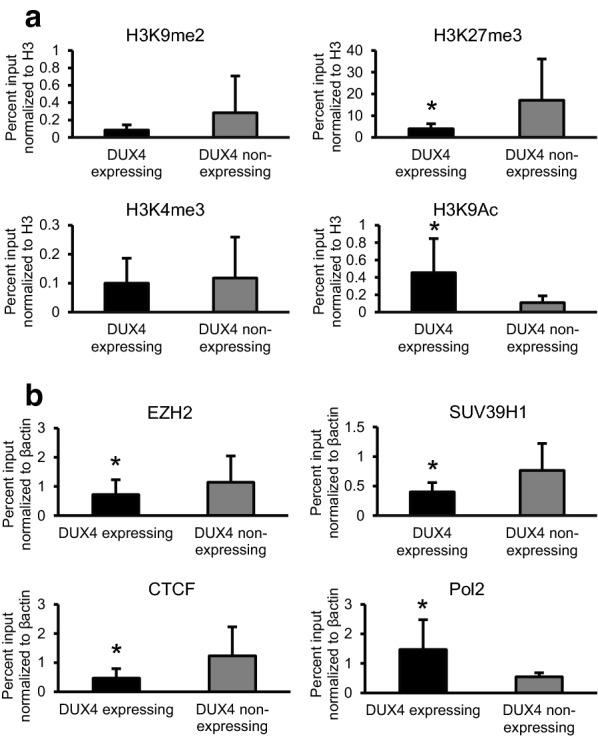
DUX4 expressing cells have increased H3K9 acetylation and decreased H3K27me3. **a** Differentiated myocytes from a FSHD1-affected individual were sorted into DUX4 expressing and non-expressing populations using a fluorescent DUX4-target reporter. Antibodies against inhibitory (H3K9me2, H3K27me3) and activating (H3K4me3, H3K9Ac) histone modifications were used to compare differences in modification levels between DUX4 expressing and non-expressing cell populations from the same culture. Percent input normalized to H3 is shown on the Y axis. **b** The levels of chromatin modifiers, EZH2 (member of the PRC2 complex that methylates H3K27), SUV39H1 (involved in H3K9 methylation) and structural protein CTCF were measured at the permissive contracted D4Z4 array and compared in DUX4 expressing and non-expressing FSHD-affected differentiated myocyte populations. Values shown are the percentage of signal obtained from input chromatin normalized to β-actin. Error bars show standard deviations of 6–12 replicates. Signal was determined by specific PCR amplification of the permissive allele using the LLP primers (see Fig. 1). The presence or absence of RNA polymerase 2 (Pol2) was used as a positive control. Statistical comparison was performed using *t* test with *p value ≤ 0.05 as being significant

CpG methylation analysis (Fig. 2) suggested that there are other epigenetic marks that distinguish DUX4 expressing and non-expressing D4Z4 arrays. We sorted DUX4 expressing and non-expressing myocytes from the same individual and screened D4Z4 associated histones for epigenetic modifications that might distinguish DUX4 expressing and non-expressing cells. Analysis of myogenic differentiation markers MYH1, MYH2 and MYOG revealed similar gene expression levels between the DUX4 expressing and non-expressing myocytes (Additional file 1: Figure S3) demonstrating that DUX4 expressing and non-expressing cells have similar differentiation profiles. Surprisingly levels of H3K4me3 and H3K9me2 modifications were similar between DUX4 expressing and non-expressing cells (Fig. 4a). However, histones containing H3K27me3 were fourfold more abundant on D4Z4 arrays that were not expressing DUX4 (Fig. 4a). In addition, histones containing H3K9 acetylation modifications were ~ fourfold more abundant at D4Z4 in DUX4 expressing cells (Fig. 4a). These findings demonstrate that even short D4Z4 arrays are under repressive epigenetic pressure despite the aberrant D4Z4 array length and suggest that repressive mechanisms and signals are intact in these cells but sporadically fail in a small population of cells.

### Chromatin modifications and mediators that suppress DUX4 transcription

Lysines at position 27 on the histone 3 tail are methylated by polycomb repressive complex 2 (PRC2), a complex of proteins that contains 4 main subunits (EZH2, SUZ12, EED and RbAp46/48) (for review see [26]). We found similar levels of EZH2 association with D4Z4 in non-FSHD control and FSHD-affected myoblasts (Fig. 3c). However, we found an increase in EZH2 association at D4Z4 in DUX4 non-expressing cells again correlating with an increase in H3K27me3 modifications in DUX4 non-expressing cells and suggesting that PRC2 recruitment is a component of the repressive machinery at contracted pathogenic D4Z4 arrays (Fig. 4b). Antibody to RNA polymerase 2 was used as a positive control to indicate RNA transcription at the DUX4 expressing LLP locus. These results suggest that PRC2 is important for repression of DUX4 expression in the absence of H3K9me3 and CpG methylation and likely does not depend on H3K9me3 or CpG methylation for recruitment.

A separate group of proteins including SUV39H1 mediates the methylation of H3K9 which in turn recruits HP1γ and the cohesion complex [27–29]. This complex is primarily active at repetitive DNA such as pericentromeric and telomeric heterochromatin, the latter being the location of FSHD-causing D4Z4 arrays. Although previous studies have shown that association of this complex with D4Z4 is highly reduced in the context of both FSHD1 and FSHD2 [16, 17], immunoprecipitation of SUV39H1 demonstrated that these proteins are preferentially associated with D4Z4 in DUX4 non-expressing cells, and therefore may also participate in the maintenance of DUX4 suppression in the context of FSHD (Fig. 4b).

CTCF binding has been shown to be regulated by histone methylation and nucleosome occupancy over CTCF binding sites. CTCF can mediate transcriptional silencing or activation by creating accessible or inaccessible loops of chromatin at specific sites [30]. We found CTCF to be more readily associated with transcriptionally silent arrays (Fig. 4b) suggesting CTCF also plays a role in repressing DUX4 transcription. Likewise, another structural component of chromatin and obvious candidate for transcriptional regulation of DUX4 is SMCHD1 [31]. Mutations in SMCHD1 result in D4Z4 CpG hypomethylation and set up cellular conditions that result in sporadic expression of DUX4 without the need for very short D4Z4 array contractions [32]. While SMCHD1 activity is central for DUX4 silencing, SMCHD1 levels were similar when comparing transcriptionally active and silenced arrays in FSHD1 myoblasts (data not shown). This observation suggests a role distinct from CTCF where SMCHD1, like “array length,” is involved in stabilization of silent arrays so that sporadic DUX4 expression does not occur, but once destabilized, SMCHD1 appears to be minimally involved in the decision of whether DUX4 transcription is activated or repressed.

### Validation of ChIP findings by chemical inhibition of chromatin modifiers

**Fig. 5:**
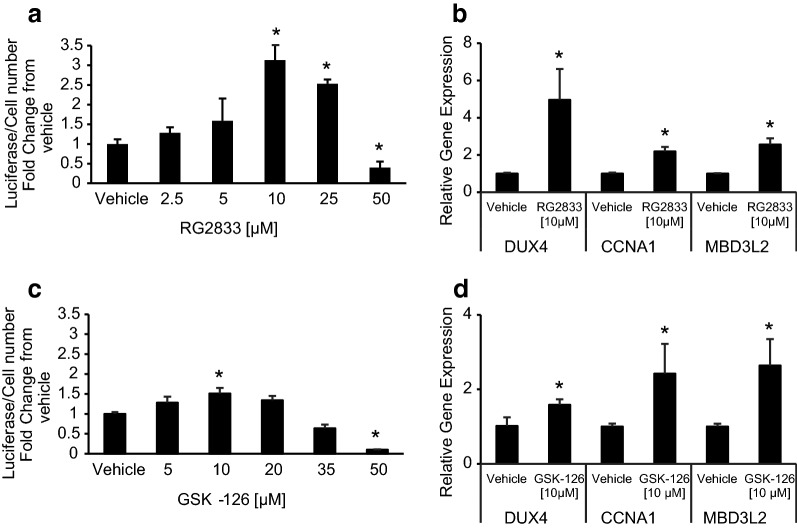
DUX4 and DUX4-target gene expression in response to chemical inhibition of chromatin modifiers. Dose escalations of the HDAC inhibitor RG2833 (**a**) or the EZH2 inhibitor GSK-126 (**c**). Cultured myoblasts were treated with the vehicle or the indicated drug at the time differentiation is initiated. Luciferase output from a DUX4-reporter is measured 48 h later, and cell numbers are estimated using the fluorescence signal from CellTiterFluor. Shown is the fold change in luciferase output normalized to cell number when the treatment is compared to vehicle alone. Analyses of all samples were performed in quadruplicate. Reductions in luciferase signal seen at higher drug concentrations (RG2833 25–50 µM and GSK-126 20–50 µM were a consequence of decreased myoblast fusion). Gene expression of DUX4 and its secondary targets CCNA1 and MBD3L2 for RG2833 at 10 µM concentration (**b**) and GSK-126 at 10 µM concentration (**d**) are shown. RNA expression was normalized to GAPDH (for DUX4) and RnaseP (for CCNA1 and MBD3L2) as endogenous controls. Analyses of all samples were performed in triplicate. *p* values were calculated using the Student’s *t* test to compare vehicle and the treated group with *p ≤ 0.05 as being significant

We screened several chemicals known to inhibit enzymes involved in histone modification to validate our ChIP results and determine whether any could be used to reduce DUX4 expression. Our finding that histone acetylation promotes DUX4 expression suggests that histone deacetylase (HDAC) inhibitors should augment DUX4 expression and histone acetyltransferase (HAT) inhibitors may suppress DUX4 expression. We selected HDAC inhibitor RG2833 (RGFP109) [33], and multiple HAT inhibitors (CPTH2 [34], garcinol [35], and C646 [36]) and treated FSHD myocytes containing a DUX4 Luciferase reporter with increasing doses while measuring DUX4 activity and cell viability. As expected, HDAC inhibition with RG2833 resulted in increased DUX4 expression as well as expression of DUX4 target genes CCNA1 and MBD3L2 in a dose-dependent manner (Fig. 5a, b).  Higher doses (> 10 µM) inhibited fusion and reduced the natural amplification of DUX4 target expression so apparent reductions of luciferase activity at higher doses are an artifact of this fusion dependent assay. DUX4 expression was reduced by treatment of cells with chemicals that inhibit HAT, however, cell viability and/or differentiation was concomitantly reduced so we were unable to conclude whether HAT inhibition directly affects DUX4 transcription.

The finding that H3K27me3 modifications are a distinguishing epigenetic mark for DUX4 expressing and non-expressing arrays suggests that chemicals that inhibit H3K27 methylation should increase DUX4 expression in an FSHD1 context. PRC2 facilitates methylation of H3K27 and GSK-126 [37] is a specific chemical inhibitor of the EZH2 component of PRC2. Consistent with these findings, previous studies have shown an increase in DUX4 expression from FSHD2 myoblasts treated with GSK-126 [38] and here we treated FSHD1 myoblasts with GSK-126 and observed a dose-dependent increase in DUX4 expression and DUX4 targets from differentiated myoblasts further validating our ChIP findings (Fig. 5c, d). Higher doses (>10 µM) inhibited fusion and reduced the natural amplification of DUX4 target expression so apparent reductions of luciferase activity at higher doses are an artifact of this fusion dependent assay. 

## Discussion

Other groups have compared epigenetic marks at D4Z4 in FSHD myoblasts with D4Z4 epigenetic modifications in non-FSHD control myoblasts. These studies revealed a clear reduction in H3K9me3 at D4Z4 in people with FSHD and also show differences in H3K9me3-dependent D4Z4 association of HP1γ, cohesion, and SMCHD1 [16, 17]. Despite these established differences, most FSHD myoblasts are indistinguishable in their transcription profile when compared to non-FSHD control myoblasts including the lack of differences in expression of DUX4 and its downstream targets [13]. Therefore, while D4Z4 chromatin changes clearly poise a muscle cell for deleterious expression of DUX4, additional events are required for the initiation of DUX4 expression.

Using fluorescence from a DUX4 reporter, we previously measured the frequency of DUX4 expression in cultured primary myoblasts and differentiated myocytes from 5 different individuals and repeated this experiment with differentiated myocytes from 2 individuals in this study (Additional file 1: Figure S1). We found that the fraction of DUX4 expressing cells varied significantly from patient to patient and ranged from 0.26% in one individual to as high as 4.28% in another. Regardless of the absolute levels, this survey demonstrated that significant transcriptional repression is occurring in > 95% of cells despite the clear genetic and epigenetic differences between FSHD and control cells [13]. By carefully selecting myoblast cells from people with unique genotypes, we have characterized several epigenetic differences between DUX4 expressing and DUX4 non-expressing pathogenic D4Z4 arrays present in FSHD myocytes. This approach exhibits some important differences from previous epigenetic studies. Here we were able to measure transcription from a single allele, and perform ChIP studies on the same allele without contaminating signal from other very similar D4Z4 sequences present on the non-pathogenic D4Z4 array on chromosome 4 and the non-pathogenic D4Z4 arrays on chromosome 10 and elsewhere in the human genome.

**Fig. 6:**
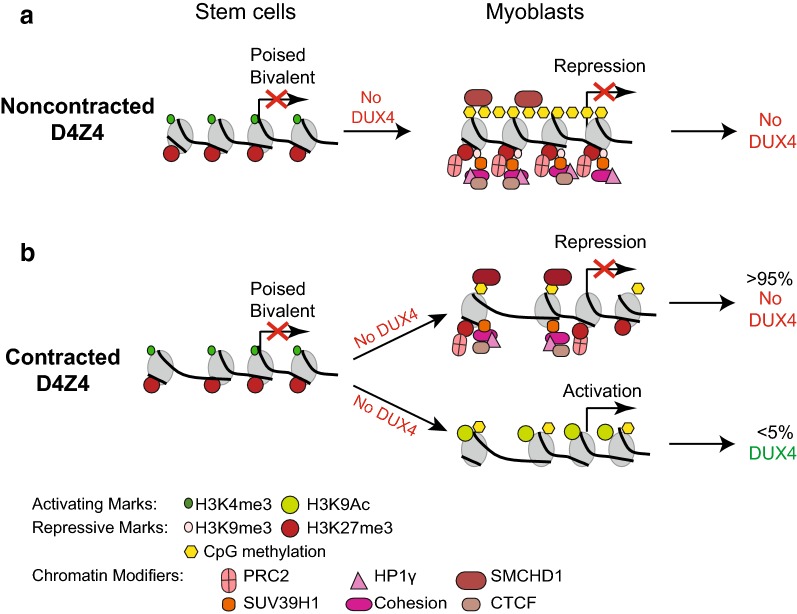
Increase in activating H3K9Ac mark leads to DUX4 expression in a small fraction of cultured FSHD myoblasts. The epigenetic landscape in human iPS and ES cells in the non-contracted (**a**) and contracted (**b**) D4Z4 locus start out as bivalent and become univalent upon differentiation into myoblasts. Even in the euchromatin state majority of the cells with the permissive D4Z4 arrays do not express DUX4 but a small fraction of cells express DUX4 due to specific activating histone mark

The enzymatic mediators of repressive chromatin modifications can be divided into two classes. Members of the first class (PRC2, SUV39H1) place H3K27me3 and H3K9me3 modifications at D4Z4, the latter facilitating binding of HP1γ and CTCF and are involved in silencing DUX4 expression from arrays that are hypomethylated at CpG residues due to contractions in length (FSHD1) or SMCHD1 deficiency (FSHD2). While H3K9me3 at D4Z4 has been shown to be reduced in FSHD [16], H3K27me3 levels are not significantly different when comparing D4Z4-associated histone modifications in FSHD and control cells [17, 38, 39]. Our results show differential labeling of histones with H3K27me3 when comparing DUX4 expressing and non-expressing cells from the same individual indicating that this histone modification may be important for transcriptional repression in the absence of H3K9me3 modification and associated HP1γ and cohesion complexes. Removal of the H3K27me3 repressive marks, along with H3K9 acetylation, results in DUX4 expression (Fig. 6). These observations are consistent with newly reported results showing the nucleosome remodeling deacetylase (NuRD) complex is important for DUX4 repression in unaffected cells and H3K9 acetylation could possibly be antagonized by HDAC1 and HDAC2 components of the NuRD complex [9]. The observation that the majority of short pathogenic chromosome 4 arrays are silenced suggests that augmenting PRC2 activity may reduce DUX4 expression further and be a worthwhile therapeutic strategy for disease treatment.

## Conclusions

Our findings both extend and clarify the findings of others. DUX4 transcription in FSHD myoblasts appears to be fundamentally due to a defect in the association of a group of chromatin modifiers whose function is to stabilize repressive chromatin structure and make repressed but poised D4Z4 arrays no longer easily activated through covalent and structural modifications of the DNA. Members of this group of epigenetic modifiers require epigenetic modifications initiated by SUV39H1 (H3K9me3) and HP1γ/cohesion association and include DNMT3b [40] an enzyme involved in methylating CpG residues, and SMCHD1 [32] a structural molecule involved in organizing higher-order chromatin structure [41, 42]. A previous study also suggests that LRIF1 may facilitate the interaction of SMCHD1 with HP1γ [41, 43] The activities of these molecules and perhaps others create structural chromatin changes that prevent the “leaky” sporadic DUX4 expression seen in FSHD due to incomplete repression by the PRC2 complex.

## Methods

### Ethics statement

Myoblast and fibroblast from individuals with FSHD and non-FSHD control myoblast from de-identified human biopsies were provided by the Fields Center for FSHD Research biorepository (https://www.urmc.rochester.edu/neurology/fields-center/research-info/sharingbiologicalresources.aspx) and utilized in this study. The non-FSHD control fibroblast and A9chr10 (hybrid) cells were obtained from the Coriell Institute for Medical Research with repository numbers GM05387 and GM11688, respectively. This study was performed in accordance and approval of the University of Washington Institutional Review Board.

### Generation of isogenic iPS cell clones and identification of array sizes

Fibroblasts from an individual with FSHD were genetically tested and were identified to have somatic mosaicism for the D4Z4 array size on chromosome 4. Human iPS cell clones were generated from this individual’s fibroblasts using the protocol of Takahashi et al. [44]. Individual clones were isolated and screened for their D4Z4 array sizes by hybridizing the p13E11 probe to Southern blots of DNA fragments separated by pulse field gel electrophoresis as described previously [45] and according to the protocol posted on the website for the Fields Center for FSHD Research (https://www.urmc.rochester.edu/fields-center/research-info/protocols.aspx).

### Cell culture

Myoblasts were cultured in F10 medium (Life Technologies, Carlsbad, CA) supplemented with 20% fetal bovine serum (Thermo Scientific, Waltham, MA), 10 ng bFGF (Life Technologies), 1 µM dexamethasone (Sigma) and 1 × penicillin/streptomycin (Life Technologies). For differentiation the proliferating myoblasts were plated at a seeding density of 70,000 cells/cm^2^ and cultured with or without 1 mM EGTA in DMEM: F12 (1:1, Life Technologies) supplemented with 20% KOSR (Life Technologies) and 1 × penicillin/streptomycin (Life Technologies) for 48–72 h.

Human stem cells were cultured on irradiated mouse embryonic fibroblasts in media containing F12/DMEM (Life Technologies), 20% knockout serum replacer (Life Technologies), 1 × penicillin/streptomycin (Life Technologies), 1% 100 mM sodium pyruvate (Life Technologies), 1 × non-essential amino acid (Life Technologies), 0.1% 0.1 M β-mercaptoethanol (Sigma) and 2 ng/ml FGF-2 (Life Technologies). Stem cell colonies were separated from irradiated mouse embryonic fibroblast layers using dispase (Life Technologies) and seeded to matrigel-coated dishes in mTESR1 media (Stemcell Technologies) and cultured at 37 °C in 5% oxygen and 5% CO_2_.

### Flow cytometry

FSHD myoblasts (see “Cell Culture” section) expressing CDK4 and transduced with the reporter vector [13] were differentiated in the presence of 1 mM EGTA to inhibit myoblast fusion and enzymatically detached from culture dishes using 0.05% trypsin-EDTA (Life Technologies). The cells were centrifuged, and the pellet was resuspended in PBS containing 1% fetal bovine serum (Thermo Scientific). The cells were sorted on a four-laser Aria II cell sorter, and data were analyzed using FlowJo at the University of Washington Cell Analysis Facility. GFP+ and GFP− cell populations were collected. These sorted cells were utilized for the bisulfite and Matrix ChIP assays.

### DNA CpG methylation analysis of LLP

DNA methylation was analyzed by bisulfite conversion (EpiTect Bisulfite Kit, Qiagen) of genomic DNA from differentiated myocytes. To perform the bisulfite analysis of the 4qA-L allele, previously published [21] PCR oligonucleotide primers (BSS4qALF for forward 5′-TTATTTATGAAGGGGTGGAGTTTGTT and BSS3626R for reverse 5′-AACAAAAATATACTTTTAACCRCCAAAAA) were utilized. All PCRs were performed using the hotstart Taq (JumpStart REDTaq from Sigma) at 95 °C for 5 min, 95 °C for 30 s, 55 °C for 30 s, 40 cycles at 72 °C for 1 min and 72 °C for 1 min. The amplified 354-bp PCR product was then gel purified and cloned into pGEM-T-Easy vector system II (Promega) and transformed into DH10B electrocompetent cells. The DH10B cells that carried the ligated vectors were selected by color change on agar plates containing the ampicillin/X-gal/IPTG. The white colonies represented the vectors with the PCR product, while the blue colonies represented empty vectors. The white colonies were picked and bulked in LB medium. The plasmids containing the target DNA were extracted by using the QIAprep Spin Miniprep Kit (Qiagen) and sequenced to reveal methylation distribution at a single molecule level.

### Chromatin immunoprecipitation

#### Sequential and single ChIP

Adherent human iPS cells and myoblasts were enzymatically detached, centrifuged, washed in PBS and cross-linked with 1% formaldehyde for 20 min at room temperature. The cells were then incubated in 125 mM glycine at room temperature for 5 min to inhibit cross-linking. The resultant chromatin was washed in PBS and suspended in a low-detergent shearing buffer containing 150 mM NaCl, 50 mM Tris, pH = 7.5, 5 mM EDTA, 0.005% NP-40, 0.01% Triton X-100 and a cocktail of protease inhibitors. The chromatin was sheared for 2 cycles of 15 min each in a bioruptor to achieve DNA fragments of < 500 bp. A total of 2 × 10^6^ cells were utilized per sequential ChIP or for input DNA. The sheared chromatin was incubated with the first antibody in an ultrasonic bath for 60 min at 4 °C. The chromatin-antibody mixture was then centrifuged, and the top 90% of the supernatant was pipetted and incubated in a protein A-agarose (Sigma) slurry at 4 °C for 45 min. The slurry was washed 4 times with cold immunoprecipitation buffer containing 150 mM NaCl, 50 mM Tris-HCl (pH 7.5), 5 mM EDTA, NP-40 (0.5% vol/vol), Triton X-100 (1.0% vol/vol) and lastly with TE buffer (10 mM Tris pH 8, 1 mM EDTA). The slurry was eluted in 30 mM DTT, 500 mM NaCl, 0.1% SDS by incubating at 37 °C for 20 min [24]. The second antibody was added to the eluate and incubated in an ultrasonic bath for 60 min at 4 °C, followed by incubation with protein A-agarose and washed 4–5 times with cold immunoprecipitation buffer. Chromatin + antibodies and ethanol-precipitated input DNA were boiled in a slurry containing 10% Chelex-100 resin. The chromatin was further incubated with proteinase k at 55 °C for 30 min and then boiled for 10 min to remove protein contaminants. The chromatin was centrifuged, and the resulting supernatant was used for real-time PCR. Single ChIP experiments were performed with the above protocol without the addition of the second antibody. This protocol was adapted and modified according to previously published method for fast ChIP [46]. The antibodies used were H3K27me3 (Abcam: ab8580) and H3K4me3 (Abcam: ab6002). Analyses of all samples were performed in triplicate.

#### Matrix ChIP

ChIP was performed on differentiated myocytes utilizing a previously published microplate-based chromatin immunoprecipitation method (Matrix ChIP) [47]. Briefly, 96-well microplates with reactin-bind protein A (Pierce) were incubated with protein A on a low-speed shaker at room temperature overnight. The next day, the wells were blocked with blocking buffer containing 5% BSA and immunoprecipitation buffer on a shaker at 40 °C for 60 min. Simultaneously, chromatin samples (see sequential ChIP to obtain chromatin) with blocking buffer and antibody were added to a new UV-modified polypropylene 96-well microplates (Genemate) and incubated in ultrasonic bath for 60 min at 4 °C. The blocking buffer was aspirated from the protein A-coated plate, and the chromatin + antibody mix was added to the wells and incubated in the ultrasonic bath for 60 min at 4 °C. The chromatin samples were washed 3 times with immunoprecipitation buffer and then TE buffer. Finally, elution buffer containing 25 mM Tris base, 1 mM EDTA (pH10) with proteinase K 200 µg/ml was added to the wells, then shaken for 30 s at 1400 rpms and incubated for 45 min at 55 °C and then 10 min at 95 °C. The 96-well plates were then briefly agitated and centrifuged for 3 min at ~ 500 g at 4 °C and were used for PCR. The antibodies utilized for Matrix ChIP were H3K4me3 (Abcam: ab12209), H3K9me2 (Abcam: ab1220), H3K27me3 (Aviva: OOAAH00064), H3K9Ac (ab 4441), Pol2 (Santa Cruz: SC47701), EZH2 (Active Motif: 39103), SUV39H1 (Aviva: 32471), CTCF (Cell Signaling: 28995), and H3 (Abcam: ab1791). Matrix ChIP experiments were performed in triplicate followed by qPCR in 6–12 replicates.

The primer sets utilized for the ChIP assays were POU4F3: Forward-TGCTGCAAGAACCCAAATTC and Reverse-GTTCTGGGCGACATGAAAAA, DUX4 LLP: Forward-CGACGGAGACTCGTTTGG and Reverse-GCTTTTGACCGCCAGGAG, HOXA3: Forward-ATGTGGCTCTTGGCTTCTCA and Reverse-GCGCATTTTTGACCCAGTTA, DIR1: Forward-CTGGGAGAATGTGCTCAGGT and Reverse-GCCAGGATTGAACAGAGGAA, β-actin: Forward-AGCACAGCCTGGATAGCAAC and Reverse-TCTGAACAGACTCCCCATCC, Forward-TCTCCCTCCTCCTCTTCCTC and Reverse-TCGAGCCATAAAAGGCAACT for Matrix ChIP.

### Drug screening and generation of the NanoLuc reporter and delivery

PCR amplified reporter cassette containing the 6 individual sequence motifs of the DUX4 DNA binding site sequence [48] AGATAATTGAATCATGGGGT AATCCAATCATGGAGTAATTTAATCAGCCGTTAATTGAATCATGG GGTAATCCAATCATGGAGTAATTTAATCAGCCG followed by a minimal TATA box promoter upstream of the NanoLuc (Promega pNL1.2[NlucP] Vector) was cloned into the pRRLsincPPT-wpre third-generation lentivirus backbone [49]. A neomycin resistance gene under the control of pTK promoter was cloned downstream of NanoLuc to establish G418 selection independent of reporter activation by the DUX4 protein. HEK-293T cells were utilized to package the lentiviral vector by polyethylenimine-mediated (PEI) co-transfection. FSHD myoblasts expressing CDK4 were then transduced with the lentivirus reporter similar to our previously published study [13].

### Cell viability assay

The FSHD myoblasts transduced with the NanoLuc reporter were seeded in 384-well plates at a cell density of 4000–7000 cells per well in proliferating media along with the drug or DMSO (vehicle). After 24 h, the media were switched to differentiating media containing the drug or DMSO. After 48 h CellTiterFluor assay (Promega) was used to measure cell viability through fluorescent signal output on a BioTek multi-detection plate reader.

### Luciferase assay

Luminescence was detected in the same cells through the Nano-Glo Luciferase assay (Promega) to measure Luciferase output, a measure of DUX4 expression. The cell-based assays were performed in quadruplicate.

### RNA preparation and qRT-PCR analysis

Adherent cells from the vehicle and drug-treated groups were lysed using 1 ml of TRIzol Cell Lysis Reagent (Life Technologies) at room temperature for 10 min. Chloroform was added at one-fifth volume of TRIzol reagent to separate the RNA containing aqueous phase from the TRIzol. The RNA was precipitated with 0.5 ml isopropyl alcohol from the aqueous phase. The RNA pellet was washed with 75% ethanol and allowed to air dry for 5–10 min and resuspended in RNAase-free water. The RNA sample was then incubated with DNAse I (NEB, Ipswich, MA) at 37 °C for 15 min followed by a cleanup step using the RNeasy column Qiagen kit. One microgram of DNAsed RNA was first primed with oligo dT primers and reverse-transcribed to cDNA using Superscript III First-Strand Synthesis System incubated at 65 °C for 5 min, followed by 50 °C for 50 min and 85 °C for 5 min. The cDNA was further diluted 1:4 to perform qRT-PCR using Roche Fast Start Universal SYBR Mastermix with ROX (Roche, Basel, Switzerland). The primer sets utilized for gene expression analysis were as follows DUX4: Forward-CTCCCGACACCCTCGGACAGCAC and Reverse-TCCAGGTTTGCCTAGACAGCGTC and GAPDH: Forward-GTGAAGGTCGGAGTCAAC and Reverse-TGAGGTCAATGAAGGGGTC. Pre-validated Taqman probes were utilized to analyze gene transcription of DUX4-activated secondary targets CCNA1 (Hs00171105_m1) and MBD3L2 (Hs00544743_m1) and RNAseP (4403326). Previously published cycling parameters were used (12) to perform the qRT-PCR using the ABI-7900HT machine. The gene expression analysis was performed in triplicate utilizing the 2^−ΔΔCt^ method.

### Statistical analyses

Student’s *t* test was utilized to calculate the statistical differences between the groups. A *p* value ≤ 0.05 was considered to be statistically significant and was denoted by asterisks. The error bars denote standard deviation.

## Additional file

**Additional file 1: Figure S1.** Quantification of DUX4 expression in FSHD cell cultures. Flow cytometry analysis of GFP fluorescence intensity on x-axis and autofluorescence on the y-axis of non-FSHD control and 2 FSHD cell lines transduced with DUX4-activated GFP reporter. **a** Proliferating myoblasts and **b** Myocytes differentiated for 48 h in differentiating media containing 1mM EGTA. **Figure S2.** H3K27me3 and H3K4me3 marks on the D4Z4 LLP locus of the isogenic iPSC clones with non-contracted and contracted D4Z4 array. Single ChIP pull down with H3K27me3 alone or H3K4me3 alone revealed the presence of both marks in the iPS cells. **Figure S3.** Similar gene expression levels of myogenic differentiation markers in DUX4 expressing and DUX4 non-expressing FSHD myocytes. Previously published and unpublished RNA-seq data obtained from Rickard et al. [13] were plotted above. Counts normalized gene expression levels were similar for MYH1, MYH2 and MYOG (markers for myogenic differentiation) with p values > 0.05 using *t*-test between DUX4 expressing and non-expressing myocytes from **a** 2349 FSHD line and **b** 2084 FSHD line.

## Abbreviations

FSHD: facioscapulohumeral muscular dystrophy
LLP: long last partial
iPS cells: induced pluripotent stem cell
ChIP: chromatin immunoprecipitation
