# Supplementary material for: Sporadic DUX4 expression in FSHD myocytes is associated with incomplete repression by the PRC2 complex and gain of H3K9 acetylation on the contracted D4Z4 allele
Source: Epigenetics Chromatin. 2018 Aug 20;11:47. doi: 10.1186/s13072-018-0215-z (PMC6100714; doi:10.1186/s13072-018-0215-z)
Supplement: Supplementary file 1 — Additional file 1: Figure S1. Quantification of DUX4 expression in FSHD cell cultures. Flow cytometry analysis of GFP fluorescence intensity on x-axis and autofluorescence on the y-axis of non-FSHD control and 2 FSHD cell lines transduced with DUX4-activated GFP reporter. a Proliferating myoblasts and b Myocytes differentiated for 48 h in differentiating media containing 1mM EGTA. Figure S2. H3K27me3 and H3K4me3 marks on the D4Z4 LLP locus of the isogenic iPSC clones with non-contracted and contracted D4Z4 array. Single ChIP pull down with H3K27me3 alone or H3K4me3 alone revealed the presence of both marks in the iPS cells. Figure S3. Similar gene expression levels of myogenic differentiation markers in DUX4 expressing and DUX4 non-expressing FSHD myocytes. Previously published and unpublished RNA-seq data obtained from Rickard et al. [13] were plotted above. Counts normalized gene expression levels were similar for MYH1, MYH2 and MYOG (markers for myogenic differentiation) with p values > 0.05 using t-test between DUX4 expressing and non-expressing myocytes from a 2349 FSHD line and b 2084 FSHD line. [file 13072_2018_215_MOESM1_ESM.pdf]

Supplementary Material

Sporadic DUX4 expression in FSHD myocytes is associated with incomplete repression by the PRC2 complex and gain of H3K9 acetylation on the contracted D4Z4 allele

Premi Haynes, Karol Bomsztyk and Daniel G. Miller

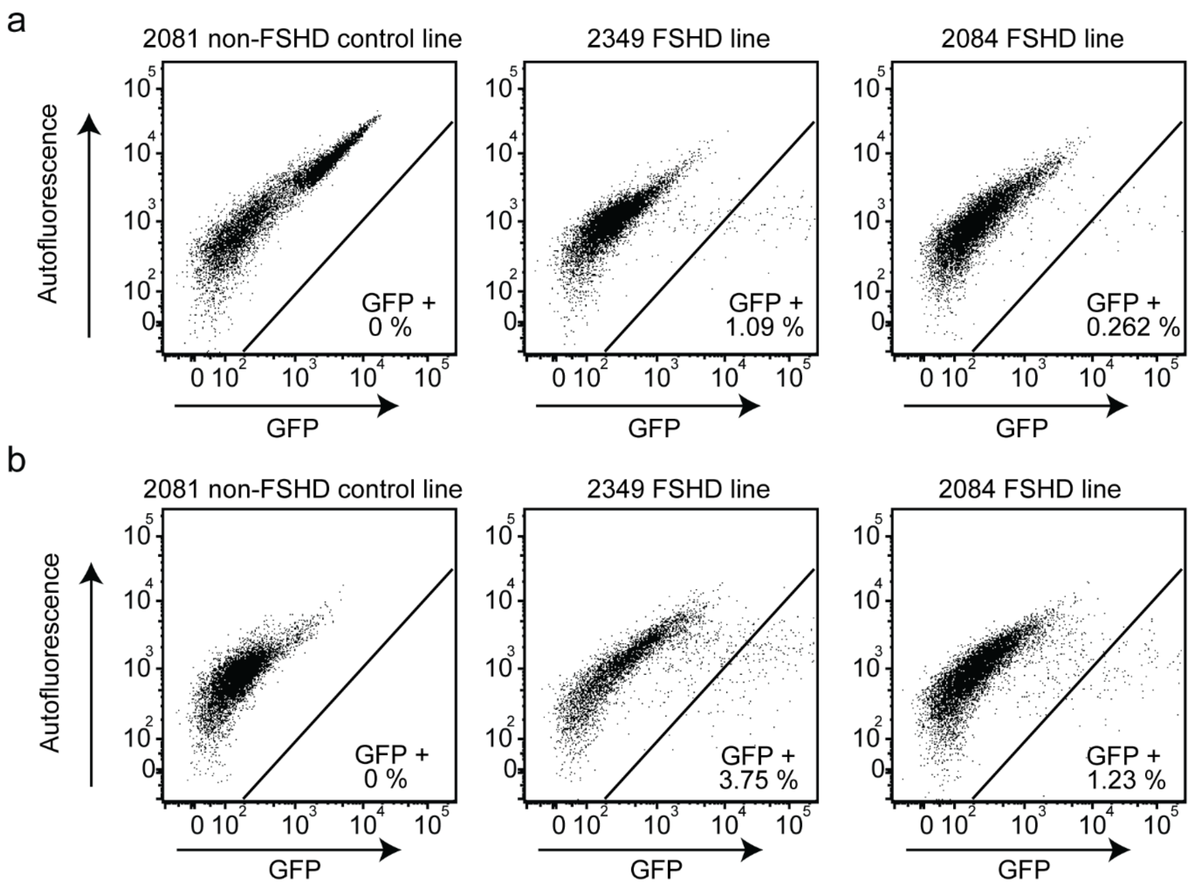

Figure S1. Quantification of DUX4 expression in FSHD cell cultures. Flow cytometry analysis of GFP fluorescence intensity on the x-axis and autofluorescence on the y-axis of non-FSHD control and 2 FSHD cell lines transduced with a DUX4-activated GFP reporter vector. a) Proliferating myoblasts and b) Myocytes differentiated for 48 hours in differentiating media containing 1mM EGTA.

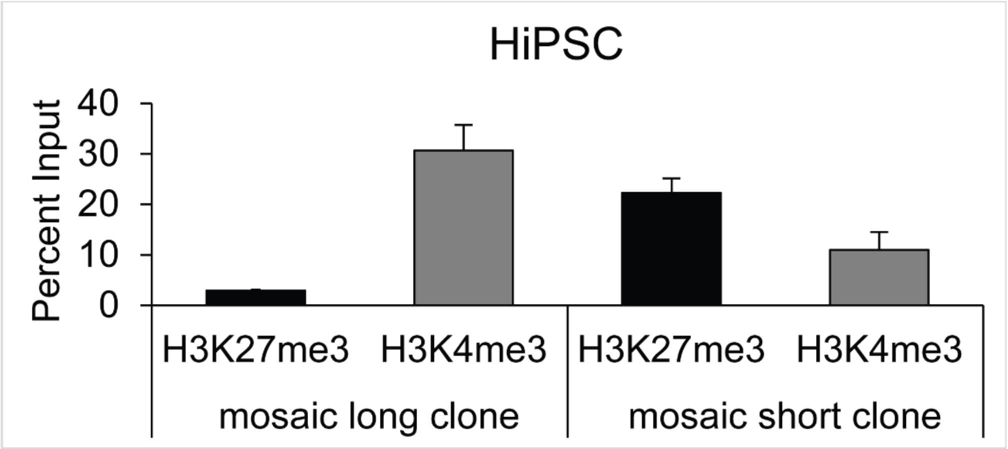

Figure S2. H3K27me3 and H3K4me3 marks on the D4Z4 LLP locus of isogenic iPSC clones with non-contracted and contracted D4Z4 arrays. Single ChIP pull down with antibodies recognizing H3K27me3 alone or H3K4me3 alone revealed the presence of both marks in the iPS cells.

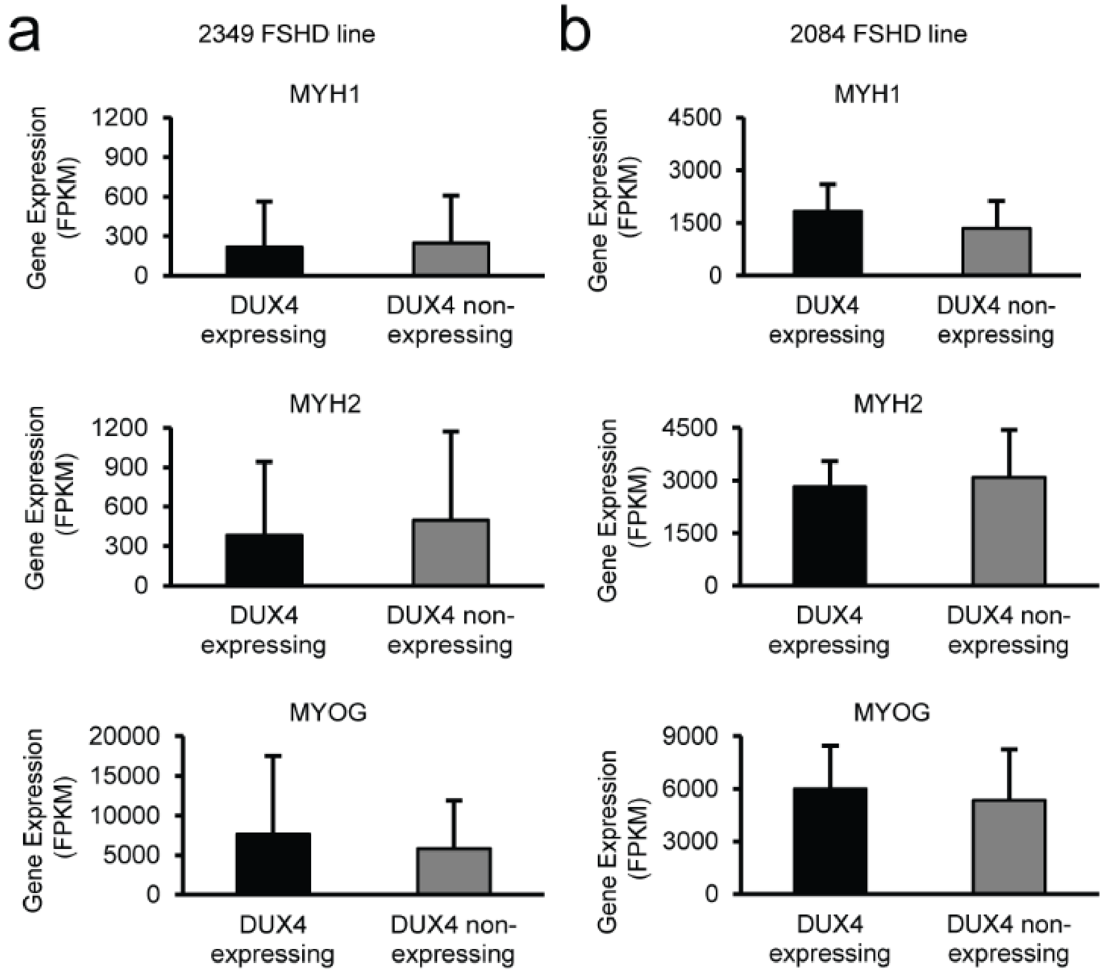

Figure S3. Similar gene expression levels of myogenic differentiation markers in DUX4 expressing and DUX4 non-expressing FSHD myocytes. Gene expression levels were similar for MYH1, MYH2 and MYOG (markers for myogenic differentiation) with p values < 0.05 using Student's t-test and comparing DUX4 expressing and non-expressing myocytes from two different FSHD-affected individuals.
